# Nachsorge für frühverwaiste Eltern in der Neonatologie: Eine Versorgungslücke der professionellen Begleitung wird geschlossen

**DOI:** 10.1055/a-2788-5417

**Published:** 2026-02-03

**Authors:** Esther Schouten, Isabella Stern, Andreas Flemmer, Teresa Starrach

**Affiliations:** 1 27192 Neonatologie, Kinderklinik und Kinderpoliklinik im Dr. von Haunerschen Kinderspital, LMU Klinikum, München, Germany; 2 27192 Klinik und Poliklinik für Frauenheilkunde und Geburtshilfe, Klinikum der LMU München, München, Germany

**Keywords:** neonatale Palliativmedizin, Trauerbegleitung, Nachsorge, neonatal palliative care, bereavement care, parental support

## Abstract

Die Überlebensrate von schwerkranken Neu- und Frühgeborenen hat sich zum Ende des letzten Jahrhunderts durch enorme medizinische Fortschritte deutlich verbessert. In den letzten Jahrzehnten wurde hierbei jedoch eine Stagnation verzeichnet. Somit ist die neonatale Palliativversorgung nach wie vor ein wichtiger Bestandteil der Betreuung kranker Früh- und Neugeborener in der Neonatologie. Das Risiko, eine psychische Erkrankung zu entwickeln, ist für verwaiste Eltern mit unverarbeiteter Trauer erhöht. Familien sind nach dem Verlust ihres Neugeborenen somit besonderen Belastungen ausgesetzt. In Deutschland gibt es bisher noch keine flächendeckende strukturierte, von der versorgenden Klinik angebotene, aufsuchende Nachsorge für frühverwaiste Eltern. Dies steht im Gegensatz zu dem Angebot, das Eltern von kranken Früh- und Neugeborenen gesetzlich zusteht. Um die bestehende Versorgungslücke von stationärer zu ambulanter Begleitung zu schließen, wurde 2019 an unserer Neonatologie ein SupportteAm für frühVerwaistE Eltern (Save) etabliert. Wir präsentieren hier einen ersten Erfahrungsbericht mit diesem neuen Programm.

## Abstract (en)

Thanks to enormous medical advances, the survival rate of seriously ill newborns and premature babies improved significantly at the end of the last century. However, stagnation has been observed in recent decades. Neonatal palliative care therefore remains an important part of care for seriously ill preterm and newborn infants in neonatology. Parents who experience the loss of a newborn are at an increased risk of developing mental health disorders. As a result, bereaved families face unique emotional and psychological challenges. To date, Germany has not implemented a comprehensive, structured, hospital-initiated, outreach-based aftercare service for parents experiencing early bereavement. This contrasts with the support services provided to parents of ill or premature newborns during and after hospital care. In order to address this gap in care and bridge the gap between inpatient support and outpatient follow-up, the Support Team for Early Bereaved Parents (SAVE) was established at our institution in 2019. Here, we report on our experience of running this new program in its first years.

## Hintergrund

Die Überlebensrate von schwerkranken Neu- und Frühgeborenen hat sich zum Ende des letzten Jahrhunderts durch enorme medizinische Fortschritte deutlich verbessert 1 . In den letzten Jahrzehnten wurde jedoch eine Stagnation verzeichnet. Somit ist die neonatale Palliativversorgung nach wie vor ein wichtiger Bestandteil der Betreuung kranker Früh- und Neugeborener in der Neonatologie. Im Jahr 2024 verstarben in Deutschland 1 645 Kinder während der Neonatalperiode und darüber hinaus sind ca 2 900 Totgeburten dokumentiert 2 . Eltern, die sich gleichzeitig mit dem Begrüßen und dem Abschiednehmen von ihrem Kind auseinandersetzten müssen, erleben große Überforderung und Hilflosigkeit 3 . Die Trauer um das eigene Kind wird als extrem intensiv und langanhaltend beschrieben. Zudem bedingt der Bruch der natürlichen Reihenfolge, nach der Kinder ihre Eltern üblicherweise überleben, den Trauerprozess negativ 4 . Das Risiko eine psychische Erkrankung zu entwickeln ist für verwaiste Eltern mit unverarbeiteter Trauer erhöht. Hierzu zählen Angststörungen, Depressionen, Somatisierungsstörungen und komplizierte Trauerverläufe 5 . Aufgrund der reduzierten Lebensqualität nehmen diese Eltern häufiger medizinische Leistungen in Anspruch 6 7 . Eine dänische Studie zeigte darüber hinaus, dass die Suizidrate betroffener Mütter in den ersten drei Jahren nach dem Verlust eines Kindes doppelt so hoch ist, wie in einem Vergleichskollektiv 8 .

Familien sind nach dem Verlust ihres Neugeborenen somit besonderen Belastungen ausgesetzt. Gleichzeitig entsteht der Eindruck, dass das soziale Umfeld die Nöte frühverwaister Eltern möglicherweise nicht ausreichend antizipieren und adäquat auf sie reagieren kann. Häufig kam der Tod des Kindes plötzlich und der Familie nahestehende Personen konnten es in vielen Fällen nicht kennenlernen. Die Existenz des Kindes bleibt daher für das Umfeld irreal. Eltern berichten, dass Freunde und Bekannte mit unbeholfenen Kommentaren auf ihre Trauer reagieren oder sich gar zurückziehen 9 10 . Der Prozess der Bewältigung eines solchen Verlustes wird von verschiedenen Faktoren beeinflusst. Festzuhalten ist, dass sich die Qualität und Intensität von Fürsorge, Unterstützung und Verständnis, die während der Trauer erfahren werden, auf das gesamte weitere Leben der Betroffenen auswirken 11 .

Kliniken in den USA bieten sogenannte „bereavement programs“ als Trauerverarbeitungsangebote an. Diese entsprechen einem Nachsorgeprogramm mit kurz- oder mittelfristiger Unterstützung betroffener Familien. Es konnte gezeigt werden, dass Eltern, die an einem solchen Programm teilnehmen, weniger psychische Belastung empfinden 12 13 . Interessant ist außerdem, dass ein besonders positiver Effekt beobachtet werden konnte, wenn die Trauerbegleitung durch die Mitarbeiter*innen der betreuenden Klinik gestaltet wurde 14 . Eltern profitieren dabei davon, dass die gemeinsame Verantwortung von Klinik und Eltern auch nach dem Tod fortgeführt wird 3 .

## Fallbeispiel

Eine 34-jährige Erstgravida mit einer risikolosen Einlingsschwangerschaft. Die Betreuung der Schwangerschaft erfolgte entsprechend der Mutterschaftsrichtlinien und war stets unauffällig. In 39+5/7 SSW stellte sich die Schwangere mit vorzeitigem Blasensprung und Unterbauchschmerzen im Kreißsaal vor. Aufgrund eines pathologischen CTGs bei Kreißsaal-Aufnahme wurde eine Notsektio in Intubationsnarkose durchgeführt, intraoperativ zeigte sich eine vorzeitige Plazentalösung.

Geboren wurde ein schwer deprimiertes Neugeborenes (NA-pH 6,98, APGAR 2/3/6, Geburtsgewicht 3370 g), das mit Beatmung und Kreislaufunterstützung zunächst stabilisiert werden kann. Aufgrund der schweren Aspyhxie wurde eine therapeutische Hypothermiebehandlung begonnen. Nach 24 Stunden entwickelte das Kind therapieresistente Krampfanfälle, im Ultraschall zeigt sich eine beidseitige raumfordernde intracerebrale Blutung (IVH Grad III). In der Folge sistiert die Eigenatmung des Kindes. Nach ausführlichen Gesprächen mit den Eltern erfolgte gemeinsam die Entscheidung zur Therapiezieländerung.

Die Familie wird nun vom neonatologischen interdisziplinären Team fortwährend sehr eng begleitet, mit dem Ziel, so viel Zeit wie gewünscht als Familie zu dritt verbringen zu können und gleichzeitig zu keiner Zeit das Gefühl zu haben, alleingelassen zu werden. Ein interdisziplinäres Team aus Ärzt*innen, Pflegekräften und Psycholog*innen sorgt für Zugang zu verschiedenen Ritualen, um Erinnerungen zu schaffen und hält gemeinsam mit den Eltern das Unaushaltbare aus. Das Kind verstirbt in den Armen des Vaters. Am Folgetag gehen die Eltern mit einer leeren Babyschale und unerfüllter Hoffnung nach Hause. Dort trifft sie die Trauer und der Schock über das Erlebte mit voller Wucht. Alles ist still. Alles anders als noch in der Klinik. Angehörige kommen vorbei und bringen, neben lieben Worten und Verpflegung, ihre eigene Trauer mit. Die Familie berichtet sechs Wochen später bei einem Nachgespräch in der Klinik davon, dass etwas fehlte. Dass mit dem nach Hause kommen eine Lücke in der professionellen Begleitung entstand. Die Eltern waren zu diesem frühen Zeitpunkt mit ihrer Trauer und gleichzeitig der Suche nach ambulanten Unterstützungsmöglichkeiten für Sterneneltern überfordert.

## Diskussion

In Deutschland gibt es bisher noch keine flächendeckende, strukturierte und von der versorgenden Klinik angebotene aufsuchende Nachsorge für frühverwaiste Eltern. Dies steht im Gegensatz zu dem nahezu flächendeckenden Angebot, das Eltern von kranken Früh- und Neugeborenen erhalten. Diese können nach dem Verlassen der Klinik auf eine sozialmedizinische Nachsorge nach § 43 Abs. 2 SGB V zurückgreifen. Das Ziel dieser Nachsorge definiert der Bundesverband Bunter Kreis e.V. folgendermaßen: „[B]erat[ung] und [B]egleit[ung] im Übergang von der intensiven Klinikbetreuung nach Hause. Sie leistet damit einen wichtigen Beitrag zur nachhaltigen Stabilität der betroffenen Familien. Im Mittelpunkt der Nachsorge stehen neben der medizinischen Versorgung des erkrankten Kindes auch die Familie, das Beziehungssystem und die Lebensumwelt“. Frühverwaiste Eltern könnten, gleichsam wie Eltern von kranken Früh- und Neugeborenen, von dem Angebot einer gesetzlichen sozialmedizinischen Nachsorge profitieren. Betroffene Familien benötigen in vielen Fällen Unterstützung bei der Stabilisierung ihrer Lebensumwelt und ihres Beziehungssystems. Darüber hinaus bleibt das Thema Bindung zu ihrem Kind auch nach dem Tod relevant und zeigt sich durch den Verlust und möglicherweise vorangegangene Sorgen deutlich erschwert (vgl. Leitsätze für Palliativversorgung und Trauerbegleitung in der Peri- und Neonatologie) 15 .

Immer wieder berichteten in der Vergangenheit betroffene wie im obigen Fallbeispiel von einer Lücke in der professionellen Begleitung unmittelbar nach dem Verlassen des Krankenhauses nach dem Kindstod. Um diese Versorgungslücke von stationärer zu ambulanter Begleitung zu schließen, wurde 2019 an unserer Neonatologie ein **S** upportte **A** m für früh **V** erwaist **E** Eltern ( *Save* ) etabliert.

Im Folgenden soll dieses Programm erläutert werden. *Save* könnte eine Blaupause für eine Nachsorgestruktur an anderen Perinatalzentren darstellen und langfristig als Kassenleistung, ähnlich der sozialmedizinischen Nachsorge, angeboten werden.

### Ein Nachsorgeprogramm für frühverwaiste Eltern

Das definierte Ziel von *Save* ist es, frühverwaisten Eltern und deren Familien eine professionelle, lokale, flexible und aufsuchende Nachsorge mit dem Schwerpunkt Trauerbegleitung anzubieten und dies über den regulären Klinikaufenthalt der Eltern hinaus. Momentan erhalten alle Eltern, deren Kind auf unserer Neonatologie verstirbt das Angebot dieser Nachsorge.

Die Erarbeitung des Konzepts für *Save* erfolgte in Zusammenarbeit mit dem Team der Elternberatung der Charité Berlin, die im Rahmen der stationären Elternberatung bereits über ein gut etabliertes, auf die Klinik begrenztes, Angebot der Trauerbegleitung verfügen. Die Nachsorgestruktur *Save* überschreitet die Grenze der Klinik und begleitet die Eltern bis nach Hause. Zur Etablierung wurden Spenden über den Förderverein der Neonatologie FrühStart ins Leben e.V. akquiriert.

### Personal und Finanzierung

Eine Gruppe aus vier Fachgesundheits- und Kinderkrankenpfleger*innen bildet das Kernteam von *Save* , welches die Begleitung der Familien übernimmt. Diesem steht ein Beratungsteam ( **siehe Infobox** ) zur Verfügung. Alle Pflegekräfte absolvieren Weiterbildungen in Trauerbegleitung sowie in Palliative Care. Die Eltern profitieren dabei emotional davon, dass die *Save* -Pflegekraft das Kind und die Familie bereits in der Klinik kennenlernen konnte. Viele Eltern verspüren eine enge Verbindung zu dem Personal, dass ihr Kind lebend kennenlernte, insbesondere, da der Kreis dieser Personen häufig klein ist.

*Save* ist aktuell eine rein spendenfinanzierte Initiative. Jährlich werden hierfür neue Spenden akquiriert. Für betroffene Eltern entstehen keinerlei Kosten. Die Pflegekräfte werden über eine geringfügig entlohnte Beschäftigung bezahlt und versichert. Die steuerlichen und rechtlichen Vorgaben eines „Minijobs“ müssen eingehalten werden. Der Stundenlohn orientiert sich an dem Stundenlohn, den Pflegefachkräfte in der sozialmedizinischen Nachsorge erhalten. Die logistische Abwicklung der Gehälter wird von der Frühgeborenen-Nachsorgeeinrichtung Dr. von Haunersche Nachsorgeeinrichtung (HaNa) in Kooperation mit dem Landesverband Bayern für körper- und mehrfachbehinderte Menschen e.V. (LVKM Bayern) übernommen.

### Ziele und Ablauf der Nachsorge

Das Ziel von *Save* ist es, Eltern in einer Phase zu begleiten, die meist von Gefühlen der Hilflosigkeit und Überforderung geprägt ist. Über einen Zeitraum von 10–12 Wochen wird der Bedarf an längerfristiger Trauerbegleitung durch vor Ort etablierte Trauerorganisationen ermittelt und wenn gewünscht bei der Vernetzung unterstützt.

Die Begleitung verläuft flexibel und wird mit den betroffenen Familien individuell abgestimmt. Dabei orientiert sie sich jedoch an einem vorgegebenen Gerüst, das unter anderem ein Aufnahmegespräch und ein gegenseitiges Kennenlernen mit der betreuenden Save-Pflegekraft umfasst. Zudem gehört dazu auch die gemeinsame Begegnung mit dem verstorbenen Kind oder den verstorbenen Kindern. Die Nachsorge beinhaltet außerdem (sozialrechtliche) Beratungen, beispielsweise zu Mutterschutz, Kindergeld, Elterngeld oder Rückbildung. Weiterhin wird die Begleitung bei Terminen unterstützt, etwa bei Besuchen beim Bestattungsunternehmen und es erfolgt eine Vernetzung zu ambulanten Hilfsangeboten, wie Selbsthilfegruppen. Das Angebot erhalten die betroffenen Familien mündlich und mit Hilfe eines Flyers noch auf der Station durch das betreuende ärztliche bzw. psychosoziale Team. Wenn Eltern das Nachsorge Angebot annehmen, erfolgt meist ein erstes Kennenlernen mit der betreuenden Save-Pflegekraft noch auf Station, wobei ein Folgetermin vereinbart wird.

**Abb. 1:**
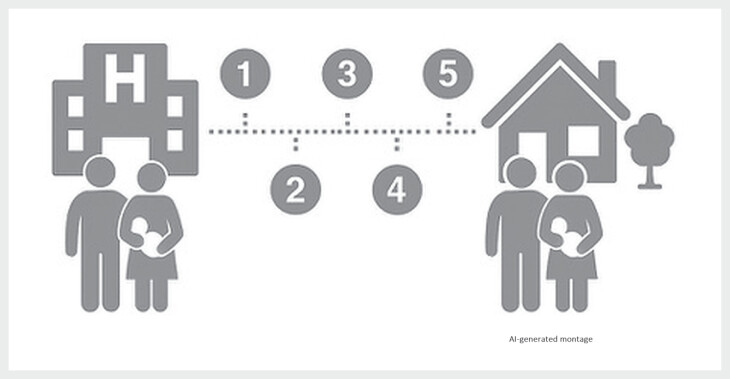
Organisation sozialmedizinischer Nachsorge bei Frühverwaisten Eltern im Save Programm. 1. Information an den Eltern über das Angebot von Save. 2. Schweigepflichtentbindung einholen. 3. Zuständige Pflegekraft für die Familie wird bestimmt. 4. Übergabe von psychosozialem Team an zuständige Save-Pflegekraft. 5. Vereinbarung erster Nachsorge Termin mit den Eltern (Quelle: Abbildung mit Chat GPT Version 5.1 am 28.11.2025 erstellt [rerif]).

Die praktische Umsetzung der Begleitung ist in Abb. 1 (erstellt mit Hilfe von künstlicher Intelligenz, Chat GPT) schematisch dargestellt. Während der Begleitung entscheiden die Eltern selbst, ob die Termine persönlich, telefonisch oder über Videokonferenz stattfinden sollen. Die bisherigen Erfahrungen zeigen, dass die Familien persönliche Termine bevorzugen. Ein Termin dauert dabei ca. 90 Minuten. In gleicher Weise bleibt zunächst auch die Frequenz, in der die Kontakte stattfinden offen und wird individuell abgestimmt. Im Schnitt haben sich Treffen alle ein bis zwei Wochen etabliert. Die Frequenz nimmt im Laufe der Begleitung meist etwas ab. Im Fokus steht neben oben genannten Themen die emotionale (Trauer-)Begleitung der Eltern. Bei den Terminen bietet die *Save* -Pflegekraft zudem erweiterte Methoden an, um Erinnerungen zu schaffen („Memory-Making“), z. B. kreative Arbeiten für das Grab oder die Erinnerungsschatulle. Neben den beiden Elternteilen nehmen immer wieder auch Geschwisterkinder und vereinzelt erweiterte Familienmitglieder, wie bspw. Großeltern, an den Save Terminen teil.

Die Arbeit mit frühverwaisten Eltern wird von den betreuenden Save-Pflegekräften als gleichsam sinnstiftend und herausfordernd beschrieben. Um dem Team ausreichend Möglichkeiten zur Reflexion anbieten zu können, finden jährlich drei Supervisionen sowie regelmäßige Intervisionen statt. Während den laufenden Begleitungen ist zudem ein regelmäßiger Austausch zwischen der Save-Pflegekraft und dem Beratungsteam möglich, der regelhaft in Anspruch genommen wird.

In den letzten fünf Jahren seit Etablierung wurde die Begleitung im Rahmen des *Save* -Programms 25 Familien nach dem Tod ihres Kindes im Kreißsaal oder auf der neonatologischen Intensivstation angeboten. Bisher wurden 18 Familien betreut, die Rückmeldungen sind durchweg positiv. „Offen über den Verlust sprechen können“, „gemeinsame Erinnerungen teilen“‚ „Rückhalt“ und „emotionale Unterstützung“ sind Kernaussagen in den Rückmeldungen von betroffenen Familien.

### Ausweitung in Kreissaal mit einem Hebammen- gestützten Team

Ein nächstes Ziel für *Save* stellt die Ausweitung des Angebotes auf Familien, deren Kind im Kreißsaal verstarb oder tot geboren wurde, dar. Vorstellbar und wünschenswert ist ein Hebammen-gestütztes Team, das die besonderen Bedürfnisse von Eltern auch nach sehr frühen Verlusten antizipieren kann und Eltern durch diese besondere Zeit begleitet. Bislang leiden Eltern nach dem frühen Verlust ihrer Schwangerschaft unter reduzierter Sichtbarkeit in der Gesellschaft. Erfreulich ist daher, dass Müttern nach Schwangerschaftsverlusten ab der 13. Schwangerschaftswoche seit Juni 2025 nun auch der Anspruch auf Mutterschutz zugesprochen wird. Ein Kreißsaal *Save* -Konzept könnte Eltern, ebenso wie das *Save* -Konzept der Neonatologie, bei behördlich-organisatorischen Fragen unterstützen, individuelle Trauerbegleitung anbieten und somit die Schwere des frühen Verlusts anerkennen.

## Fazit für die Praxis

Frühverwaiste Eltern sind in besonderem Maße gefährdet, eine psychische Störung und/ oder körperliche Erkrankung in Folge des Verlustes ihres Kindes zu entwickeln. Bislang haben die betroffenen Eltern keinen gesetzlichen Anspruch auf sozialmedizinische Nachsorge. Die Rückmeldungen der bisher durch *Save* betreuten Familien sind eindeutig: frühverwaiste Eltern können von einer entsprechenden Nachsorge profitieren. Diese sollte flexibel, individuell, lokal und kostenlos für die Eltern sein und eine Brücke zwischen der Betreuung im stationären Aufenthalt und bestehenden ambulanten Angeboten darstellen.

*Save* ist ein Beispiel für eine solche Nachsorge und kann als Blaupause fungieren, um andere Kliniken zu motivieren, eine vergleichbare Nachsorgestruktur entwickeln können. Das Ziel sollte sein, dass alle Perinatalzentren standardmäßig ein Nachsorgekonzept für frühverwaiste Eltern anbieten können und die anfallenden Kosten als Kassenleistung anerkannt werden.

*Save* versteht sich im vorgestellten Setting als Primärprophylaxe für die langfristige Gesundheit betroffener Eltern und Familien.

## Notes

Infobox
**Beratungsteam**

- Neonatolog*in
- Palliativmediziner*in
- Geburtshelfer*in/Pränataldiagnostiker*in
- Psycholog*in
- Seelsorger*in
- Sozialarbeiter*in
